# Microleakage of Class II Bulk-Fill Resin Composite Restorations Cured with Light-Emitting Diode versus Quartz Tungsten-Halogen Light: An In Vitro Study in Human Teeth

**DOI:** 10.3390/biomedicines11020556

**Published:** 2023-02-14

**Authors:** Jenny López-Torres, Karen Hernández-Caba, Luis Cervantes-Ganoza, Marysela Ladera-Castañeda, Reynaldo Martínez-Campos, Fredy Solís-Dante, Gissela Briceño-Vergel, César Cayo-Rojas

**Affiliations:** 1 School of Stomatology, Universidad Privada San Juan Bautista, Lima 15067, Peru; 2 Research Team “Salud Pública—Salud Integral”, Faculty of Dentistry and Postgraduate School, Universidad Nacional Federico Villarreal, Lima 15001, Peru; 3 Faculty of Stomatology, Universidad Inca Garcilaso de la Vega, Lima 15084, Peru; 4 Oral Rehabilitation Department, School of Stomatology, Universidad Científica del Sur, Lima 15067, Peru; 5 Faculdade do Centro Oeste Paulista, Bauru 17012, Brazil

**Keywords:** resin composite, microleakage, thermal cycling, tooth preparation, light-emitting diode, quartz tungsten-halogen

## Abstract

**Background:** Resin composites undergo a certain degree of shrinkage when light-cured with different light sources available on the market, resulting in microleakage of dental restorations. The aim of the present study was to assess microleakage of class II restorations with bulk-fill resin composites cured with LED (light-emitting diode) and QTH (quartz tungsten-halogen light) units, both in cervical and occlusal areas of cavity preparations. **Materials and Methods:** In the present in vitro experimental study, a total of 30 human molar teeth were used, in which 60 class II cavities were prepared (mesial and distal) and restored with Filtek bulk fill resin composite. Restorations were equally distributed in 3 groups according to type of curing light: A, QTH (Litex 680A Dentamerica^®^); B, LED (Bluephase N^®^ 3rd generation); and C, LED (Valo^®^ 3rd generation). Each group was further subdivided into subgroups 1 and 2 according to IV-A or IV-B resin composite color. Restored teeth were subjected to 20,000 thermal cycles between 5° and 55 °C, then immersed in 1M silver nitrate solution for 24 h. Subsequently, the teeth were sectioned mesiodistally to obtain samples for observation under stereomicroscope in order to determine microleakage degree. Kruskal–Wallis H and Mann–Whitney U statistical tests were applied with a significance level of 5% (*p* < 0.05). **Results:** No statistically significant differences were found in the degree of microleakage of bulk-fill resin composites light-cured with LED and QTH units for both occlusal (*p* > 0.05) and cervical areas (*p* > 0.05). Additionally, no significant differences were found when comparing microleakage between occlusal and cervical areas (*p* > 0.05), regardless of lamp type. In addition, significant differences in microleakage degree were found between bulk-fill resins with IV-A and IV-B shades when they were light-cured with QTH at cervical level (*p* = 0.023). However, there were no significant differences when comparing these bulk-fill resin composite shades at occlusal level with LED (*p* > 0.05) and QTH (*p* > 0.05) units. **Conclusions:** Class II restorations with bulk-fill resin composite in IV-A and IV-B shades light-cured with third generation LED lamp and QTH showed no significant differences in microleakage when compared in both occlusal and cervical areas. On the other hand, significantly more microleakage was found at the cervical level when a darker shade of resin composite was used and light-cured with the QTH unit.

## 1. Introduction

Currently, dental esthetic treatments are one of the main reasons for patient requests. In order to improve these treatments, major dental materials companies are innovating and perfecting their supplies, equipment, and restorative instruments. In this way, they aim to provide dentists the necessary alternatives to restore morphological characteristics of treated teeth, with good esthetics and optimal physical properties [1]. Among resin composites that offer these advantages are bulk-fill resins, since they allow light-curing a block of resin 4 to 5 mm deep without prolonging the exposure time to light, thus avoiding greater tension in the adhesion area [2,3,4]. In addition, the polymerization initiation system has been improved in most of the bulk-fill resin composites. Tetric N-Ceram bulk fill, for example, has a new initiator called ivocerin that has been added to its standard initiator system (lucerin and camphorquinone), which has higher reactivity compared to camphorquinone. This initiator increases the polymerization depth to 4 mm and reduces clinical working time. In addition, Tetric N-Ceram bulk fill has two prepolymers and filler particles (isofiller) that reduce shrinkage stress during polymerization [2].

Camphorquinone is activated with a wavelength ranging from 390 nm to 510 nm in intensity, with an ideal peak at 470 nm, has a yellowish color and is usually in proportion of 0.15 to 0.20% [3,4]. The absorption spectrum of ivocerin ranges from 370 nm to 460 nm, represents 0.2% of the composition of these composites, and in combination with camphorquinone achieves a more complete polymerization. Lucerin TPO has an absorption spectrum between 350 nm to 430 nm with a peak at 400 nm and has a light color often used in certain adhesives and composites of enamel or translucent shades [5].

Quartz-tungsten-halogen (QTH) lamps have been frequently used for camphorquinone polymerization. However, these types of lamps present limitations that affect the polymerization degree of resin composites such as limited lifetime of the bulb (40–100 h) and low efficiency in conversion of electrical energy to heat production with high light [6,7] in addition to emitting a wavelength that varies between 400 and 500 nm with intensity ranging between 400–600 mW/cm^2^ [8]. On the other hand, third generation LED (light-emitting diode) lamps are light-curing units with a light intensity that varies depending on manufacturer from 800 to 1500 mW/cm^2^ with a wavelength range from 395 to 515 nm and a service life of more than 10,000 h [4,9,10,11], experiencing little degradation of light output over time [6]. Difference in intensity and wavelength can be a key point to achieving optimal polymerization as complete activation of photoinitiators in the deep part of a restoration depends on it [9,11,12,13]. For example, the third-generation light-curing unit of Valo^®^ manufacture is a light-curing device that has an average light intensity of 1000 mW/cm^2^ and a wavelength range of 395 to 480 nm [10], while the Bluephase N^®^ manufacture light-curing device emits a light intensity of 1200 mW/cm^2^ and a wavelength range of 385 to 515 nm [11].

Due to wavelength emitted by LED curing units, camphorquinone activation is considerably higher than in conventional halogen curing units, resulting in a more complete polymerization [12,14]. However, due to the high power of LED curing units, they could cause a very abrupt activation, generating more stress than usual and therefore could affect the marginal seal quality of restorations, causing microleakage, which would have a negative influence on dental restorations longevity as it could be a risk factor for recurrent caries, hypersensitivity, discolorations, and pulp lesions, among others [15].

Therefore, the clinical success of a restoration depends on several factors such as depth of cure (DOC), degree of conversion, shrinkage stress, and other factors. In turn, the DOC is influenced by many factors such as the size, type, and content of the filler; the thickness and shade of the material; and the efficiency of light transmission (irradiance, exposure time, distance from the light source). In this regard, inadequate polymerization leads to a significant decrease in the physical and biocompatibility properties of resins [16].

In view of the above, the present study aimed to assess the microleakage of class II restorations with bulk-fill resin composite light-cured with LED and QTH units, both in cervical and occlusal areas of cavity preparations. Considering as null hypothesis that there are no differences in microleakage degree of class II restorations with bulk-fill resin composites cured with LED and QTH curing units both in cervical and occlusal areas of cavity preparations.

## 2. Materials and Methods

### 2.1. Type of Study and Delimitation

This experimental in vitro study was conducted at School of Stomatology, Universidad Privada San Juan Bautista, Peru, from May to June 2022, with approval letter from an institutional ethics committee No. 690-2022-CIEI-UPSJB. This research respected the bioethical principles for medical research involving human subjects of the Declaration of Helsinki related to confidentiality, freedom, respect, and non-maleficence and also considered the CRIS Guidelines (Checklist for Reporting In-vitro Studies) [17].

### 2.2. Sample Size and Selection

**Figure 1:**
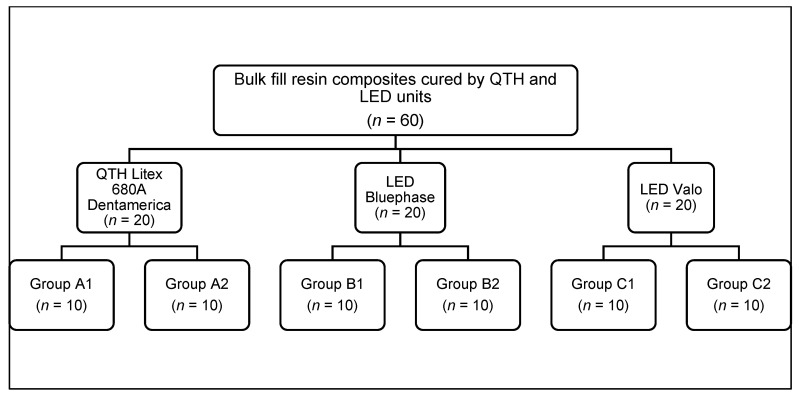
Random distribution of sample units according to type of light-curing unit.

In total, 60 class II cavities were prepared in 30 healthy human molar teeth, extracted for orthodontic purposes and donated by patients who gave informed consent to be used for research purposes. All methods were performed in accordance with relevant guidelines [18]. The sample size for each subgroup (A1, B1, C1, A2, B2, and C2) was 10 class II cavities (*n* = 10), calculated on the basis of data obtained in a pilot study with 5 sample units per group. The formula for two-tailed comparison of proportions was applied with the statistical software G*Power version 3.1.9.7 considering a significance level (α) = 0.05 and a statistical power (1-β) = 0.80, with proportions P_1_ = 0.8 and P_2_ = 0.2. The sample units were selected by simple random method without replacement and were distributed in 6 groups (Figure 1):

- A1: Mesial cavity restored with Tetric N-Ceram Bulk-Fill IV-A resin composite, light-cured with Dentamerica^®^ Litex 680A lamp.
- B1: Mesial cavity restored with Tetric N-Ceram Bulk-Fill IV-A resin composite, light-cured with Bluephase N^®^ lamp.
- C1: Mesial cavity restored with Tetric N-Ceram Bulk-Fill IV-A resin composite, light-cured with Valo^®^ lamp.
- A2: Distal cavity restored with Tetric N-Ceram Bulk-Fill IV-B resin composite, light-cured with Litex 680A Dentamerica^®^ lamp.
- B2: Distal cavity restored with Tetric N-Ceram Bulk-Fill IV-B resin composite, light-cured with Bluephase N^®^ lamp.
- C2: Distal cavity restored with Tetric N-Ceram Bulk-Fill IV-B resin composite, light-cured with Valo^®^ lamp.

### 2.3. Cavity Preparations in Teeth

**Figure 2:**
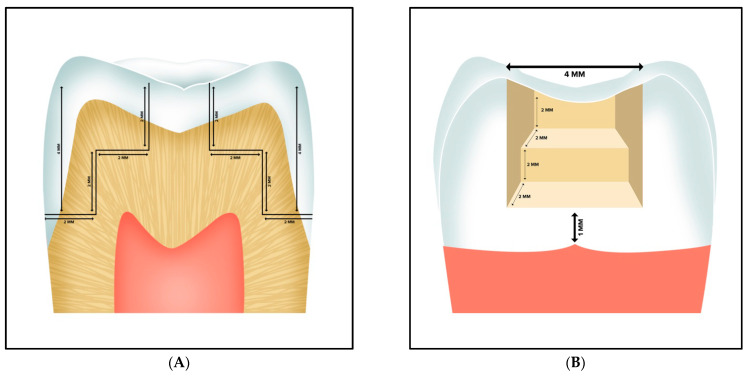
Class II cavity preparation. (**A**): crown sectioned lengthwise (mesiodistal direction) and (**B**) proximal view.

Immediately after extraction, the teeth were washed and immersed in a 1% t-chloramine solution (Milipore, Supelco, Lima, Peru) for a week for disinfection. Then, they were placed in a container with distilled water at 4 °C for maintenance for no longer than 3 months and water replacement every 7 days. Subsequently, 24 h before cavities were prepared, the teeth were placed in distilled water at 23 ± 2 °C for conditioning. All cavities and dental restorations were performed by a single operator using a high-speed handpiece with No. 1092 cylindrical diamond bur (Microdont, Sao Paulo, Brazil) and abundant water cooling. Cylindrical bur was replaced every 2 preparations. Two Black’s class II cavities were prepared in mesial and distal of teeth, with standardized measurements [15] (Figure 2). All cavities were made 1 mm above the amelocemental junction and their dimensions were checked twice by two investigators with a North-Carolina-type periodontal probe (Hu-Friedy, Chicago, IL, USA).

### 2.4. Cavity Conditioning

All cavities were conditioned with 37% phosphoric acid gel for 15 s, then washed with abundant water and dried with sterile gauze balls until dentin was wet, and then a layer of fifth generation adhesive Tetric^®^ N-Bond was placed with microbrush and air was gently applied for 5 s with a triple syringe to evaporate the solvent [19,20]. For adhesive light-curing in A1 and A2 groups, a QTH lamp was used with intensity of 700 mW/cm^2^ for 40 s; for B1 and B2 groups, a 3rd generation Bluephase LED lamp was used with intensity of 1200 mW/cm^2^ for 10 s; and for C1 and C2 groups, a 3rd generation Valo LED lamp was used with intensity of 1000 mW/cm^2^ for 10 s. Then, a circumferential metal matrix (Automatrix^®^ MT, Dentsply, Charlotte, NC, USA) was adjusted around each class II cavity to achieve an adequate conformation of the restoration walls.

### 2.5. Restoration of Dental Cavities

All groups were restored by the same operator using a single increment of 4 mm thick Tetric^®^ N-Ceram Bulk-fill resin composite (Table 1).

- Groups A1 and A2 were light-cured from occlusal for 40 s with a Dentamerica^®^ QTH Litex 680A lamp at 700 mW/cm^2^ intensity.
- Groups B1 and B2 were light-cured from occlusal for 10 s with a Bluephase N^®^ LED lamp at 1200 mW/cm^2^ intensity.
- Groups C1 and C2 were light-cured from occlusal for 10 s with a Valo^®^ 3rd generation LED lamp at 1000 mW/cm^2^ intensity.

Light intensity of the three light-curing units was verified using a radiometer (Bluephase Meter II, Ivoclar Vivadent AG, Schaan, Liechtenstein). After finishing restoration, the teeth were immersed in distilled water at 37 ± 2 °C to be incubated for 24 h. Then the same operator polished all the resin composite block surfaces with an electric motor (EM-E6, W&H, Bürmoos, Austria) and a contra-angle handpiece (NSK, Tokyo, Japan) for 20 s per step according to the manufacturer’s specification with a four-step coarse-to-superfine grain disc system (Sof-Lex, 3M ESPE, St Paul, SM, USA) at speed of 15,000 rpm with identical movements and in the same direction. The polishing discs were changed after use on each sample. Then the samples were washed to remove surface residues and allowed to dry.

### 2.6. Thermocycling and Teeth Immersion in Dye

Teeth were subjected to 20,000 thermocycles between 5 °C and 55 °C, exposure to each bath was 30 s, and transfer time between baths was 10 s [15,21]. Then, two coats of nail polish were applied 1 mm from restoration margin on the entire tooth surface and apices were sealed with light-curing glass ionomer cement (Vitrebond™ 3M ESPE, St. Paul, MN, USA) to prevent dye penetration from the apex. They were then immersed in 1M silver nitrate solution for 24 h out of reach of light and rinsed with plenty of water for 5 min. Finally, they were immersed in photorevealer solution for 8 h under fluorescent light and rinsed with abundant water.

### 2.7. Teeth Sectioning and Observation under a Stereomicroscope

The teeth roots were sectioned transversely at 3 mm below the amelocemental junction. Then, the crowns were sectioned sagittally in mesiodistal direction with a 0.20 mm thick bi-active diamond disc using a low-speed motor (Strong 210, Saeshin, Republic of Korea) with abundant water irrigation. They were allowed to dry at room temperature and observed under a binocular stereomicroscope (Leica EZ4, Wetzlar, Germany) at 20× magnification to determine the degree of dye microleakage. Stereomicroscope readings were performed by a single investigator. However, an intra-examiner calibration of the microfiltration diagnosis was performed using Cohen’s kappa index, from which 0.83 (CI: 0.51–1.00) was obtained, and this value was acceptable. A double-blind procedure was applied since the statistician and the researcher who performed the readings under the stereomicroscope were unaware of the group assignment. To measure the penetration of silver nitrate, the scoring system was used based on the technical specification ISO/TS11405:2015 [22], which mentions the following criteria:

- Score 0 (no microleakage);
- Score 1 (microleakage up to enamel);
- Score 2 (microleakage up to dentin);
- Score 3 (microleakage up to pulp floor).

### 2.8. Statistical Analysis

Microleakage results were recorded in a Microsoft^®^ Excel 2019 spreadsheet and then analyzed by the SPSS^®^ (Statistical Package for the Social Sciences) version 28.0. Because the response variable was ordinal in scale, the Kruskal–Wallis H statistical test was used to compare the microleakage of the light-cured resin composite groups with three different lamps, and the Mann–Whitney U test was used for the comparison of microleakage at the occlusal and cervical levels. In addition, the latter statistical test was used to compare microleakage of the two different shades of resin composites. A significance level of 5% (*p* < 0.05) was considered in all tests.

## 3. Results

In the occlusal area, score 0 microleakage was observed in more than 50% of the IV-A resin composite restorations cured with LED lamps. However, IV-B shade resin composites showed microleakage score 1 to 3 in more than 50% of cases. Halogen light-cured IV-A resin composite restorations showed score 0 microleakage in 50%, and the remaining 50% showed score 1 microleakage, but IV-B shades showed microleakage greater than score 0 in 70 % of the cases (Table 2). No statistically significant differences were found when comparing microleakage scores of the three curing unit groups for both IV-A and IV-B shade restorations (*p* = 0.668 and *p* = 0.656, respectively) (Table 3).

Regarding cervical area, 50 % of IV-A resin composite restorations cured with LED lamps showed score 0 microleakage, and the other 50 % showed score 1. In contrast, more than 80 % of IV-B resin composite restorations showed microleakage between scores 1 and 3. Halogen light-cured restorations showed microleakage in more than 80 % of the cases (Table 2). In addition, no statistically significant differences were found when comparing microleakage score of IV-A and IV-B shade restorations (*p* = 0.379 and *p* = 0.112, respectively) (Table 3).

When comparing microleakage in occlusal and cervical areas of each light-cured restoration group with the three lamp types, no statistically significant differences were obtained (*p* > 0.05) (Table 4).

When comparing microleakage of two shades of bulk-fill resin composite (IV-A being lighter than IV-B) according to the three light-curing units used, no statistically significant differences were obtained either in occlusal (*p* > 0.05) or cervical areas (*p* > 0.05), except for the group of light-cured restorations with QTH in the cervical area (*p* = 0.023) (Table 5).

## 4. Discussion

Microleakage is an important parameter that determines the performance of dental resin composite restorations in oral cavity, so it is important to prevent its possible causes [23]. A major problem that causes microleakage and affects the mechanical properties of resin composites is poor polymerization due to poor curing [24,25]. To improve quality of dental restorations and prevent microleakage, polymerization shrinkage should be avoided or decreased, so a high quality of light-curing should be achieved in dental restorations using an adequate system. Therefore, the present study aimed to assess microleakage of Class II restorations with bulk-fill resin composite light-cured with LED and QTH units, both in cervical and occlusal areas of a cavity preparation. Therefore, the null hypothesis was not rejected.

Different commercial brands of LED curing units have gained great popularity due to their use among professionals to achieve adequate polymerization of resin composites. There are controversial results among various studies regarding use of LED lamps. This may be due to different intensities used in the restorative protocols. In present study, for light-curing of resin composites, exposure times of 10 s with an intensity of 1000 mW/cm^2^ for the Valo lamp, 10 s with an intensity of 1200 mW/cm^2^ for the Bluephase lamp, and 40 s with an intensity of 500 mW/cm^2^ for the QTH lamp were used, as recommended by manufacturers. Most studies using these exposure times show acceptable hardness and conversion degree of bulk-fill resin composites with irradiation greater than 1000 mW/cm^2^ [26], and having a good degree of conversion increases the surface microhardness as it has been shown with a variety of different composite resins that 80% of the maximum hardness is associated with 90% of the maximum polymerization [27]. Tetric N-Ceram bulk-fill resin contains alternative photoinitiators intended to enhance photopolymerization, such as ivocerin, which are stimulated by different wavelengths, and previous studies have shown that ivocerin acts as a polymerization enhancer, allowing it to be efficient [2,15,27]. However, Alkhudhairy, in his study, reported that Tetric N-Ceram bulk-fill resin light-cured with a high intensity output (1200 mW/cm^2^) was less wear resistant and mentions that the quality of the interphase bond between the fillers and the matrix and the degree of curing of the composite resin may also influence wear resistance [28].

The present study assessed microleakage under a stereomicroscope and scanning electron microscopy was not used since our aim was not quantifying the amount of silver nitrate but the penetration of silver nitrate at the tooth–resin interface. In addition, several studies support this type of assessment, so the aim was to measure microleakage under a stereomicroscope [15,19,29,30,31].

Restored teeth were subjected to thermocycling to simulate thermal conditions in oral cavity. Not having found a specific number of cycles equivalent to physiological aging in the mouth as various authors perform this aspect according to their convenience [32]. In the present study, a process of 20,000 thermal cycles was performed since several studies suggest the parameter of 500 thermal cycles as a limited number to represent aging time according to the International Organization for Standardization (ISO) [33,34,35]. In addition, Michailesco et al. point out that 30,000 thermal cycles are equivalent to 1 year of clinical aging, while Gale et al. and Darvell et al. suggested that 10,000 thermal cycles are equivalent to the same [33,36].

In the present study, silver nitrate was used as dye since it is one of the most used in research to evaluate microfiltration and nanofiltration due to its good penetration capacity through the tooth–resin interface in addition to absorbing light by reducing the silver diamine ions to grains of metallic silver 0.059 nm in diameter, which improves its observation under the stereomicroscope. In addition, compared to methylene blue, it is not water soluble, which prevents its removal from tissue when teeth are cut using abundant cooling with water [37,38].

Some studies support that the design of tooth preparations influences the stress produced by volumetric shrinkage during light-curing of resin composites [39]. Although there is no established cavity design parameter or model for the present type of study, the tooth preparations were measured and standardized. In addition, the same procedure for acid etching and adhesive use was followed for all the samples to avoid biases in the results.

Based on results of the present study, it was found that the degree of microleakage of IV-A and IV-B shade resin composites cured with LED lamps, both in occlusal and cervical areas, was lower than those cured with a QTH lamp. However, this difference was not statistically significant, which does not exclude it from being clinically relevant. These results are in agreement with those obtained by Kumar et al. [12] and Oberholzer et al. [40], who concluded that there are no statistically significant differences in microleakage between LED and QTH lamps at the occlusal level. In addition, Nuray et al. reported that there was no significant difference between microleakage with LED and QTH lamps in cervical region [6,12,40]. The minimal differences in microleakage of halogen and LED light-cured restorations may be due to the fact that the ivocerin photoinitiator in Tetric N-Ceram bulk-fill resin composite is sensitive to short wavelengths (370–460 nm) compared to camphorquinone (390–510 nm), which acts as a photoinitiator in most conventional resin composites and bulk fills [41,42]. As ivocerin is not sensitive to wavelength peaks of a monowave LED curing light or QTH lamp, the use of polywave lamps that emit both short and long wavelengths, such as Valo and Bluephase N lamps, both third generation, is recommended for complete light-curing [8,43,44].

When comparing microleakage of IV-A and IV-B resin composite shades, no statistically significant differences were obtained between occlusal and cervical areas in any of the groups cured with LED and QTH lamps. However, the cervical restorations light-cured with QTH lamps showed significant differences between IV-A and IV-B shades, obtaining greater microleakage with the darker shade (IV-B). This could be due to the fact that the filler particle pigments affect the light transmission of the material. Such dark pigments present in the resin composite (IV-B) limit the light transmission as well as reduce the degree of conversion since a high percentage of the wavelengths, being absorbed at the surface of the composite, could not excite the co-initiators at greater depths [45]. Another possible explanation could be the low efficiency of the QTH unit in converting electrical energy into heat production [7], resulting in fewer free radicals in the deeper increments; also, considering that the IV-B color resin composite is less translucent, this probably made it more difficult for short-wavelength (violet) light to penetrate, as the resin color IV-B would need longer exposure times to achieve light penetration into deeper layers [46,47]. Because of this, perhaps the polymerization of IV-A color resin composite was more complete in contrast to IV-B color resin composite of the same trademark.

Our findings are in agreement with those of Kumar et al., who found no significant differences when comparing microleakage degree of LED cured resin composites between the occlusal and cervical margins, although they did find significantly more microleakage at the cervical margin compared to the occlusal margin when cured with a QTH lamp using the same resin composite shade [12], with the latter being in disagreement with findings of the present study. This difference may be due to the fact that Kumar et al. made cavities with cervical margins below the amelocemental boundary. In the present study the occlusal and cervical margins were limited to enamel by making the proximal box cervical margin 1 mm above the amelocemental junction as microleakage is significantly higher when cervical margin is 1 mm below the amelocemental junction [29]. This argument is reinforced by the fact that the bonding of resin composites to enamel is better than to cement as enamel has a higher inorganic composition (95%) and a lower percentage of water. In addition, cement bonding is poorer than dentine bonding because the latter has larger collagen fibers and higher hydroxyapatite volume [48,49,50].

Different curing units can influence the polymerization degree of resin composites [51]. Nassar et al. assessed the irradiance values of ten different light-curing lamps, including the LED lamps used in present study, and concluded that all devices assessed had adequate irradiance values for light-curing of resin composites [52].

In a previous study by Cayo et al. [15] where they also compared microleakage in Class II restorations with Tetric N-Ceram Bulk-Fill composite resin light-cured with Bluephase N LED lamp in human teeth subjected to 10,000 thermal cycles, they reported microleakage in occlusal with a median score of 1, compared to the present study where the median score of this group was 0, raising the question of why there would be less microleakage in the present study if subjected to 20,000 thermal cycles. These apparent discrepancies would probably not be statistically significant when comparing the median microleakage scores in both studies; considering their interquartile range, the results overlap. Under this premise, it could still be controversial why with the higher number of thermal cycles the microleakage did not increase; this could be answered by the findings reported by Michailesco et al. [30] and Gale and Darvell [33], who stated that there are no differences between 10,000 and 20,000 thermal cycles, as both cycles are equivalent to 1 year of clinical aging. Perhaps this also explains why the median microleakage at the cervical level in both studies was the same (score 1). Furthermore, the nature of the variable considered in both studies must be taken into account as the microleakage variable was worked with as an ordinal variable, and therefore non-parametric tests were used for comparisons based on the median as this measure of central tendency is not susceptible to extreme or atypical values, which may well occur in this type of measurement. However, it must be recognized that the use of this type of statistical test is a limitation as it does not allow for very precise calculations as the distribution of the data cannot be known exactly due to the type of qualitative variable [53]. Therefore, the application of parametric tests to establish differences should be considered; for future studies, to compare microfiltration, it is suggested to include quantitative variables such as the distance of dye penetration in microns through the tooth–resin interface as long as the measurements comply with the statistical prerequisites. On the other hand, another possible explanation for the discrepancies observed, which are merely descriptive, could be due to the fact that in the study by Cayo et al. [15] another adhesive system was used for the Tetric N-Ceram bulk-fill resin, since Adper Single Bond-2 was applied, while in the present study Tetric N-Bond adhesive was used, whose commercial brand is the same as the composite resin used, which could perhaps have favored a better marginal seal of the restoration in the present study. This can be supported by the report of Leon et al. [54], who compared both adhesive systems with composite resins of the same commercial brand, finding that the group of restorations where Adper Single Bond-2 adhesive was used had a higher microleakage (median = 2) compared to the group where Tetric N-Bond adhesive was used (median = 0.5); however, it should be noted that these differences were not statistically significant, as in the study by Cayo et al. [15] and in the present study. Furthermore, it should be noted that in the study by Cayo et al. [15], the Filtek bulk-fill composite resin (3M company, Saint Paul, MN, USA) showed descriptively lower median microleakage than the Tetric N-Ceram bulk-fill resin when an Adper Single Bond-2 adhesive (3M company) was used for both resins.

The present study is important because it contributes to increasing scientific evidence and allows the contrasting of these results with other authors’ results and the generation of new knowledge regarding the influence of curing-light lamps on microleakage of resin composites. In addition, to date (December 2022), there are no studies that compare microleakage in class II restorations of bulk-fill resin composites using two types of shades polymerized by different curing units, so the present study provides novel scientific evidence. These evidence-based findings will help the dental professional decide the best curing light alternative to obtain restorations with adequate polymerization and greater longevity.

### Limitations of the Study

Within the limitations of this study, it is recognized that these results should be taken with caution because, being an in vitro study design, they cannot be reliably extrapolated to clinical practice, so randomized clinical trials and more comparative studies with same aim as the present study are recommended. At the same time, it should be taken into account that in a clinical situation, temperature changes, the presence of saliva, enzymes and pH changes could affect the properties of the resin composites over time [27]. Another limitation of the present study was that we did not quantify the amount of silver ions present in the microgaps of the resin–tooth interface since a scanning electron microscope with energy-dispersive X-ray spectroscopy was not employed.

## 5. Conclusions

In summary, recognizing the limitations of the present in vitro study, it can be concluded that class II restorations with bulk-fill resin composites in shades IV-A and IV-B light-cured with third-generation LED and QTH lamps did not show significant differences in microleakage when compared both in occlusal and cervical areas. On the other hand, significantly more microleakage was found at the cervical level when a darker shade of resin composite was light-cured with the QTH unit.

## Figures and Tables

**Table 1:** Technical profile of products employed.

Product	Type	Composition	Filler % (wt-vol)	Manufacturer	Lot
Tetric ^®^ N-Ceram Bulk-Fill (IV-A and IV-B)	Nanohybrid bulk fill	Matrix: bis-GMA, bis-EMA, UDMA Filler: barium silicate aluminum glass, “isofiller” (prepolymer, glass and ytterbium fluoride), ytterbium fluoride, and mixed oxides	76 wt%—54 vol%	Ivoclar Vivadent, Schaan, Liechtenstein	Y44813/ Y39907
Etching gel Eco-Etch^®^		Phosphoric acid (37% by weight in water), thickeners, and pigments		Ivoclar Vivadent, Schaan, Liechtenstein	Y39461
Tetric^®^ N-Bond Adhesive		Phosphoric acid acrylate, HEMA, Bis-GMA, urethane dimethricrylate, ethanol, film-forming agent, indicators, and stabilizers.		Ivoclar Vivadent, Schaan, Liechtenstein	Y43470
**Light curing unit**	**Type of technology**	**Light intensity**	**Radiation time**	**Wavelength**	**Manufacturer**
QTH Litex 680A	QTH	500 mW/cm^2^	40 s	350–520 nm	Dentamerica, City of Industry, CA, USA
LED Bluephase N	LED polywave	1200 mW/cm^2^	10 s	385–515 nm	Ivoclar Vivadent, Liechtenstein
LED Valo	LED polywave	1000 mW/cm^2^	10 s	395–480 nm	Ultradent, South Jordan, UT, USA

**Table 2:** Occlusal and cervical microleakage of dental restorations with Tetric N-Ceram resin composite according to light-curing unit.

Light Curing Unit	Area	Color	Score 0		Score 1		Score 2		Score 3		Total	
			f	%	f	%	f	%	f	%	n	%
**QTH Litex 680A**	Occlusal	IV-A	5	50%	5	50%	0	0%	0	0%	10	100%
		IV-B	3	30%	4	40%	3	30%	0	0%	10	100%
	Cervical	IV-A	2	20%	8	80%	0	0%	0	0%	10	100%
		IV-B	0	0%	5	50%	4	40%	1	10%	10	100%
**LED Bluephase N**	Occlusal	IV-A	7	70%	3	30%	0	0%	0	0%	10	100%
		IV-B	5	50%	3	30%	2	20%	0	0%	10	100%
	Cervical	IV-A	4	40%	6	60%	0	0%	0	0%	10	100%
		IV-B	0	0%	9	90%	0	0%	1	10%	10	100%
**LED Valo**	Occlusal	IV-A	6	60%	4	40%	0	0%	0	0%	10	100%
		IV-B	3	30%	6	60%	1	10%	0	0%	10	100%
	Cervical	IV-A	5	50%	5	50%	0	0%	0	0%	10	100%
		IV-B	2	20%	6	60%	2	20%	0	0%	10	100%

f: absolute frequency; n: sample size; QTH: quartz tungsten-halogen lamp; LED: light-emitting diode technology.

**Table 3:** Microleakage in occlusal and cervical areas of dental restorations with Tetric N-Ceram resin composite according to area.

Area	Color	Light Curing Unit	n	Median	IQR	*p*-Value *
**Occlusal**	IV-A	QTH Litex 680A	10	0.50	1	0.668
		LED Bluephase N	10	0.00	1	
		LED Valo	10	0.00	1	
	IV-B	QTH Litex 680A	10	1.00	2	0.656
		LED Bluephase N	10	0.50	1	
		LED Valo	10	1.00	1	
**Cervical**	IV-A	QTH Litex 680A	10	1.00	0	0.379
		LED Bluephase N	10	1.00	1	
		LED Valo	10	0.50	1	
	IV-B	QTH Litex 680A	10	1.50	1	0.112
		LED Bluephase N	10	1.00	0	
		LED Valo	10	1.00	1	

n: sample size; IQR: interquartile range; QTH: quartz tungsten-halogen lamp; LED: light-emitting diode technology; * Based on Kruskal–Wallis H test.

**Table 4:** Microleakage of dental restorations with Tetric N-Ceram resin composite according to occlusal and cervical area.

Light Curing Unit	Color	Area	n	Median	IQR	*p*-Value *
**QTH Litex 680A**	IV-A	Occlusal	10	0.50	1	0.280
		Cervical	10	1.00	0	
	IV-B	Occlusal	10	1.00	2	0.165
		Cervical	10	1.50	1	
**LED Bluephase N**	IV-A	Occlusal	10	0.00	1	0.280
		Cervical	10	1.00	1	
	IV-B	Occlusal	10	0.50	1	0.165
		Cervical	10	1.00	0	
**LED Valo**	IV-A	Occlusal	10	0.00	1	0.739
		Cervical	10	0.50	1	
	IV-B	Occlusal	10	1.00	1	0.579
		Cervical	10	1.00	1	

n: sample size; IQR: interquartile range; QTH: quartz tungsten-halogen lamp; LED: light-emitting diode technology. * Based on Mann–Whitney U test.

**Table 5:** Microleakage of dental restorations according to shade of bulk-fill resin composite.

Light Curing Unit	Area	Color	n	Median	IQR	*p*-Value *
**QTH Litex 680A**	Occlusal	IV-A	10	0.50	1	0.190
		IV-B	10	1.00	2	
	Cervical	IV-A	10	1.00	0	0.023 *
		IV-B	10	1.50	1	
**LED Bluephase N**	Occlusal	IV-A	10	0.00	1	0.353
		IV-B	10	0.50	1	
	Cervical	IV-A	10	1.00	1	0.089
		IV-B	10	1.00	0	
**LED Valo**	Occlusal	IV-A	10	0.00	1	0.218
		IV-B	10	1.00	1	
	Cervical	IV-A	10	0.50	1	0.143
		IV-B	10	1.00	1	

n: sample size; IQR: interquartile range; QTH: quartz tungsten-halogen lamp; LED: light-emitting diode technology. * Based on Mann-Whitney U test.

## Data Availability

The data presented in this study are available on request from the corresponding author.
